# Supplementary material for: Alu–Mediated Duplication and Deletion of Exon 11 Are Frequent Mechanisms of PALB2 Inactivation, Predisposing Individuals to Hereditary Breast–Ovarian Cancer Syndrome
Source: Cancers (Basel). 2024 Nov 30;16(23):4022. doi: 10.3390/cancers16234022 (PMC11640139; doi:10.3390/cancers16234022)
Supplement: Supplementary file 1 [file cancers-16-04022-s001.zip › Supplementary Figure S1.pdf]

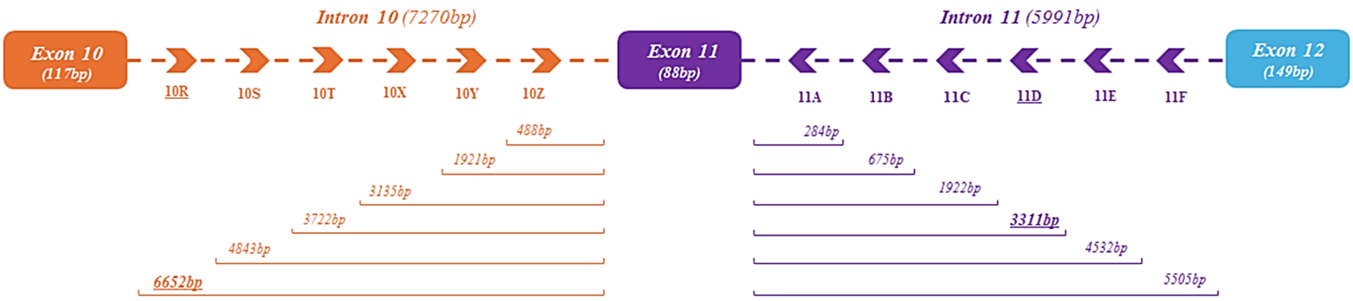

Figure S1: Schematic representation of primer walking strategy used to identify the deletion intronic breakpoints: six forward primers on intron 10 (from 10R to 10Z) and six reverse primers on intron
