# Supplementary material for: Alu–Mediated Duplication and Deletion of Exon 11 Are Frequent Mechanisms of PALB2 Inactivation, Predisposing Individuals to Hereditary Breast–Ovarian Cancer Syndrome
Source: Cancers (Basel). 2024 Nov 30;16(23):4022. doi: 10.3390/cancers16234022 (PMC11640139; doi:10.3390/cancers16234022)
Supplement: Supplementary file 1 [file cancers-16-04022-s001.zip › Supplementary Table S1.pdf]

Primer intronic position	*Primer sequences*
PALB2 I10 **R** forward	ACTTGACTTACCAACAACATCATATT
PALB2 I10 **S** forward	AATTTGACTATGTTCTGATCTGCCA
PALB2 I10 **T** forward	CAGTTACCTTGAGTTCTGTTGTACT
PALB2 I10 **X** forward	CTGTTACTTATCTTTTGGGCTCTGT
PALB2 I10 **Y** forward	CAAGGAAATGCATATGTTCTTCAGG
PALB2 I10 **Z** forward	CACTTCAATCCTTCCTTCTATCCAT
PALB2 I11 **F** reverse	ATATTGGCAAAGAGAGAATGAGGAC
PALB2 I11 **E** reverse	TTAAGAAGAGGCTGACAAACTATGG
PALB2 I11 **D** reverse	GTGGGTATTGATGTTGTAATATAAAAGGT
PALB2 I11 **C** reverse	AAGTCAGTCTCTACACAAATGCTG
PALB2 I11 **B** reverse	TGAGAAGACTGAAGCCTAAGAGTA
PALB2 I11 **A** reverse	GGCTGGGAGTCAAGTCTGA

Table S1: Sequence of each forward and reverse primer used in the primer walking strategy to identify intronic deletion breakpoints
